# Non-SARS Non-MERS Human Coronaviruses: Clinical Characteristics and Outcome

**DOI:** 10.3390/pathogens10121549

**Published:** 2021-11-27

**Authors:** Israa Saib, Saud Aleisa, Husam Ardah, Ebrahim Mahmoud, Ahmad O. Alharbi, Abdulrahman Alsaedy, Sameera Aljohani, Ahmed Alshehri, Naif Khalaf Alharbi, Mohammad Bosaeed

**Affiliations:** 1 Department of Medicine, King Abdulaziz Medical City, Riyadh 14812, Saudi Arabia; saud.naleisa@gmail.com (S.A.); emahmoud85@gmail.com (E.M.); dr.ahmad@hotmail.com (A.O.A.); SaedyAb@ngha.med.sa (A.A.); bosaeedmo@ngha.med.sa (M.B.); 2 King Abdullah International Medical Research Center, Riyadh 11481, Saudi Arabia; JohaniS@ngha.med.sa (S.A.); harbina2@ngha.med.sa (N.K.A.); 3 King Saud bin Abdulaziz University for Health Sciences, Riyadh 14611, Saudi Arabia; ardahhu@ngha.med.sa; 4 Department of Biostatistics and Bioinformatics, King Abdullah International Medical Research Center, Riyadh 11481, Saudi Arabia; 5 Department of Pathology & Laboratory Medicine, King Abdulaziz Medical City, Riyadh 14812, Saudi Arabia; ShehriA62@ngha.med.sa

**Keywords:** human coronaviruses, viral pneumonia, epidemiology, Saudi Arabia

## Abstract

Human coronaviruses (HCoVs) have become evident sources of human respiratory infections with new emerging HCoVs as a significant cause of morbidity and mortality. The common four coronaviruses (229E, HKU1, NL63, and OC43) are known to cause respiratory illness in humans, but their clinical impact is poorly described in the literature. We analyzed the data of all patients who tested positive for at least one of the four HCoVs from October 2015 to January 2020 in a tertiary care center. HCoVs were detected in 1062 specimens, with an incidence rate of 1.01%, out of all documented respiratory illnesses. Detection of these viruses was reported sporadically throughout the years, with a peak of occurrence during winter seasons. OC43 had the highest incidence (53.7%), followed by NL63 (21.9%), HKU1 (12.6%), and 229E (11.8%). Most of these infections were community-acquired, with symptoms of both upper and lower respiratory tract. Co-detection with other viruses were observed, mostly with rhinovirus. 229E was the most frequent (26.4%) HCoV in patients requiring intensive care, while NL63 and 229E were the most common in patients requiring invasive ventilation. The highest 30-day mortality rate was observed in patients infected with 229E (6.4%). HCoVs are common circulating pathogens that have been present for decades, with 229E being the most virulent in this study cohort.

## 1. Introduction

Over recent decades, it has become evident that coronaviruses cause significant morbidity and mortality in humans. There are four common coronaviruses: 229E, HKU1, NL63, and OC43 that are known to cause respiratory illness in humans with symptoms ranging from mild to severe disease. In addition, emerging coronaviruses have been documented in the past two decades. In 2002, Severe Acute Respiratory Syndrome (SARS), the first recorded human coronavirus outbreak emerged in Guangdong Province in mainland China, from where it spread to 26 countries with a total of 8,098 people worldwide becoming sick and 774 died [1,2]. A decade later, the Middle East Respiratory Syndrome (MERS-CoV) emerged in Saudi Arabia with 2468 cases and a high mortality rate of 35% [3]. By the end of 2019, Coronavirus Disease 2019 (COVID-19) caused by the novel Severe Acute Respiratory Syndrome Coronavirus-2 (SARS-CoV-2) spread worldwide and caused a pandemic with a death toll exceeding 3 million [4]. Unlike SARS-CoV, SARS-COV-2 or MERS-CoV, the common HCoVs are poorly described in the literature despite being identified for several years. The common coronaviruses are widespread in mammals, with evidence suggesting that 229E and NL63 originated from bat reservoirs, while HKU1 and OC43 emerged from rodents and domestic animals [5,6]. Previous studies have shown that OC43 had the highest incidence rate; NL63 and HKU1 have moderate prevalence; and 229E is the least prevalent [7]. Large-scale comprehensive screening for all four coronaviruses by analysis of 11,661 diagnostic respiratory samples collected in the United Kingdom over three years detected HCoVs in 0.3 to 0.85% of samples in all age groups. It showed a winter seasonality for HCoV-OC43, which was the most commonly seen coronavirus [8]. The epidemiology and clinical presentation of these coronaviruses documented from studies in different regions of the world showed that there are seasonal variation and co-association with other respiratory viruses [8,9,10,11,12,13]. A study conducted in the southwestern region of Saudi Arabia found that non-SARS non-MERS human coronaviruses were frequently detected during the winter season. OC43 was the second most common etiology in adults, after human rhinovirus, whereas HKU1 and NL63 were mostly observed in children less than 15 years old [14]. Here, we update the literature with recent data on HCoV infections in a four-year period (pre-COVID-19) from one of the largest hospitals in Saudi Arabia. This study aims to address the epidemiological and clinical characteristics of the common HCoVs in patients presenting with respiratory symptoms.

## 2. Results

A total number of 105,101 specimens from respiratory infections were examined for 17,432 patients who were suspected of having respiratory infectious illness between October 2015 and January 2020. Of these, at least one of the four common HCoVs were detected in 1062 specimens, with an incidence rate of 1.01%. Of the HCoV patients, 482 subjects were female (45%). The mean age was around 13.6 years ± 25.5, with OC43 and NL63 detected in the younger age group. The most common comorbidities were bronchial asthma (20.8%) and congenital heart diseases (23.3%), while chronic obstructive pulmonary disease (COPD) had a risk of only 3.6% (Table 1). These comorbidities were variable based on the age group. Congenital heart diseases were seen almost exclusively in the pediatric age group, 246 of 247 (99%) less than 14 years old. This was followed by bronchial asthma, as 81% (178 of 221) of asthmatic patients were less than 14 years old. Diabetes mellitus and hypertension were mainly in adults (older than 24 years), 98% (120 of 122) and 92% (122 of 133), respectively. In addition, 86% of the heart failure patients were adults, while those with an immunosuppressed status were observed across all age groups. 

**Figure 1:**
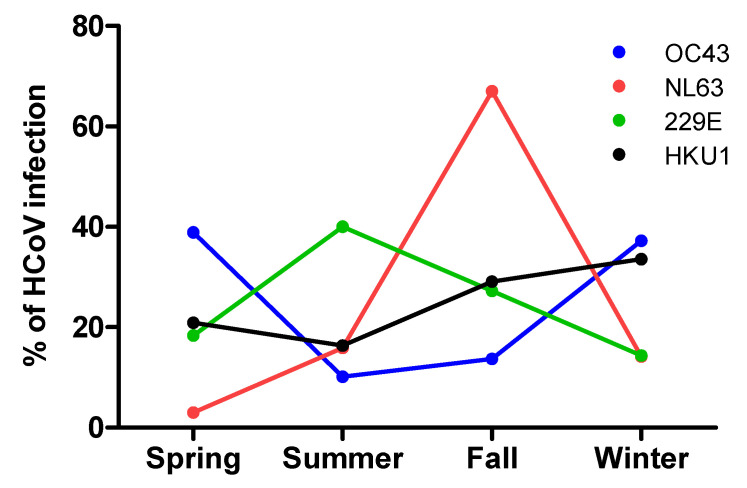
Percentage of detected HCoV infections across different seasons.

Detection of the viruses was reported sporadically throughout the year, with a peak of occurrence (29.0%; *p* value < 0.0001) from December to February (winter). The lowest incidence was documented during summer (June to August) with a detection rate of 15.7%. (Figure 1)

**Figure 2:**
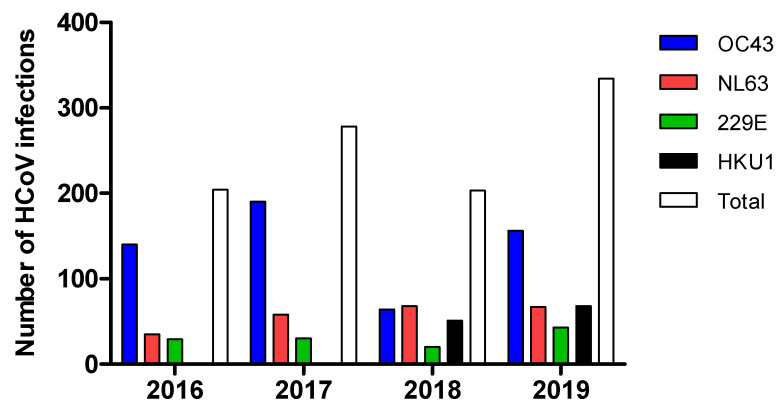
Detection of HCoVs over four years.

Persistent detection of these viruses was observed throughout the years, except for HKU1 that was not detected at all in 2016 or 2017 in this cohort. (Figure 2).

Among the studied coronaviruses, OC43 had the highest total (and annual) incidence rate (53.7%), followed by NL63 (21.9%), HKU1 (12.6%), and 229E (11.8%). Community acquired infections were the most prevalent (91.2%; *p* value < 0.0014); 36% of these cases reported contacts with influenza patients within 14 days prior to HCoV infections; and 28.3% had a history of hospitalization in the previous 30 days. Hospital acquired infections were observed in 8.8% of the cases, 3.2% of which were reported in ICU settings (Table 2). With regard to patient symptoms, fever was reported in 75.1% as the most common symptom, followed by shortness of breath (54.5%), cough (51.9%), and upper respiratory tract symptoms (45.4%) in the HCoV patients (Table 3).

Co-detection with other viruses was predominant among HCoV casesm mostly with human rhinovirus (25.3%), followed by adenovirus (14.6%), and respiratory syncytial virus (13.7%). However, a significant number of patients had solely HCoV infection (40.30%) (Table 4). Six patients in our cohort had combinations of more than one HCoV infection. Bacterial coinfections were also reported in 4.5% of the HCoV cases. *Staphylococcus aureus* was observed in 14 patients, followed by *Pseudomonas aeruginosa* in seven patients, and *Haemophilus influenza* in four patients. Other gram-negative organisms were reported as well. Secondary bacterial infections occurred after 48 hours of hospitalization in 3.2% of patients and was dominated by the gram-negative organism *Pseudomonas aeruginosa*, other cases included *Streptococcus pneumoniae* and *Staphylcoccus aureus*. 

The need for intensive care management after acquiring one of the four common coronaviruses was more frequent with 229E (26.4%), and invasive ventilation was required frequently in NL63 and 229E infected patients. The mean duration of hospitalization was around 18.8 ± 88.27 days and all-cause 30-day mortality rate was at the highest for the group infected with 229E (6.4%) (Table 5). Patients infected with OC43 had fewer ICU admissions and a lower 30-day mortality rate, 10.7% and 1.2%, respectively.

## 3. Discussion

This retrospective study reports the epidemiological spectrum of the four common human coronaviruses and their clinical characteristics in a tertiary care center over more than five years. Out of these coronaviruses, infections with OC43 were the most frequent, occurring mainly during spring and winter, followed by NL63 and HKU1; 229E infection had the lowest rate of incidence and occurred mostly during fall. Infection with 229E was the most virulent in our cohort, since it was reported more among ICU and ventilation-supported patients. Coinfections with other viruses and bacteria were not uncommon in our cohort.

Several studies have been conducted to delineate the geographical differences of Non-SARS Non-MERS coronavirus infections. OC43 and 229E were discovered in the 1960s, NL63 in 2003, followed by HKU1 in 2005. Each of these viruses was firstly isolated in different geographical areas ranging from Europe to Hong Kong [5]. This was observed in Gerna et al’s study where they reported infections with OC43-, 229E-, and NL63-like strains together with untypeable HCoV strains causing lower respiratory infections, suggesting that the number of coronaviruses causing human pathology will probably increase further [15]. The prevalence pattern in our study was in agreement with previous studies from Hong Kong, the UK, and France [7,8,16]. However, another study from Michigan, USA, noted that NL63 coronaviruses had the highest prevalence [12], which could delineate an epidemiological difference. Previous outbreaks of OC43 have been reported in Australia, France, and Canada [17,18,19], although circulation of these viruses around the world have been reported, and it is common to find one or two of the HCoVs predominantly in a certain geographical area [7,20,21,22]. 

There was a significant variation with regard to patient age. Infections with OC43 and NL63 were more detected in the pediatric group. HKU1 was more seen in young adults, while infections with 229E were encountered in adults. Similar trends of age distribution were observed in other studies [23,24]. Age variation could be related to the underlying comorbid conditions where heart diseases, either heart failure or congenital heart disease, were common risk factors for our patients. Another observation is that immunosuppressed patients were mostly infected with 229E (*p*-value < 0.0001), which is supported by previous studies [8,15,25]. The majority of HCoV infections were community-acquired rather than hospital-acquired, of which 3.2% were in the ICU. 229E was the most frequent in the ICU patients with a prolonged hospital stay. This finding denotes the role of hospitalization as a dependent risk factor for developing HCoV infections.

With regard to the clinical spectrum of these infections, most of our patients presented to the emergency department with symptoms including fever and the usual association of either productive cough, upper respiratory tract symptoms, or shortness of breath. Nevertheless, coinfections were detected in the cohort subjects, which could have partially contributed to symptoms in both lower and upper respiratory tracts. However, single infections had almost similar clinical profiles to coinfections, which would argue against the presumed benign nature of these viruses. In support of this, Gaunt et al. observed a more severe pattern of infection in cases of sole infections than in mixed infection [8]. Coinfection of human rhinovirus, adenovirus, and respiratory syncytial viruses were the most dominant in our study. The cause of viral coinfection varied in previous studies, but human rhinovirus [12,23] and respiratory syncytial virus [9] were frequently observed. Six patients had infection with a combination of more than one of the four common HCoVs, which agrees with a recent report [23]. However, the causative viruses differed, suggesting that HCoVs are common copathogens in respiratory illnesses. Bacterial coinfections are usually concurrent with HCoV infections, mostly caused by *Staphylococcus aureus* [26], and this is well-supported by our study. 

Concerning clinical outcomes, intensive care management post-infection was required for 14.8% of the patients, with more than half of those requiring mechanical ventilation. This deterioration might not be directly related to the HCoVs, rather the patient’s primary condition. The calculated all-cause 30-day mortality rate was around 2.3% with 229E carrying most of the burden. Of note, patients who died from 229E infection were mostly immunocompromised with frequent prior hospitalizations, which should alert physicians to the importance of keeping this virus in mind when managing such cases. HCoVs could be used as a prognostic marker, and not necessarily a causative factor, for the unfavorable outcome in high-risk populations. The comparison of alpha- and beta-coronaviruses reveals notable genomic features of SARS-CoV-2, which might explain the disease burden and severity. This includes the basis of structural studies where SARS-CoV-2 appears to be optimized for binding to the human receptor ACE2, and the spike protein of SARS-CoV-2 has a functional polybasic (furin) cleavage site at the S1–S2 boundary through the insertion of 12 nucleotides [27].

The present study has a limitation of being retrospective as well as not being able to include asymptomatic HCoV infections in the general population. Such asymptomatic cases require a further large-scale prospective epidemiological study to properly characterize these viruses, especially at the community level. Nonetheless, the presented data show that HCoVs are important respiratory pathogens, and hospital surveillance and effective infection control programs should consider more caution dealing with these viral infections. 

In conclusion, human CoVs are common circulating pathogens in community and hospital settings. OC43 is the most prevalent, and HKU1 has become more detectable in recent years, while 229E is the most virulent type of HCoV. Continuous evaluation of these coronaviruses is needed to better understand their epidemiology and clinical significance.

## 4. Materials and Methods

*Study design and setting:* Retrospective analysis was performed on data collected from all patients who tested positive for at least one of the four common HCoVs (229E, HKU1, NL63, and OC43). Lab tests were performed for symptomatic patients presenting to the emergency department with complaints of fever, dry cough, shortness of breath, or upper respiratory tract symptoms, such as sore throat or runny nose. Collected data included demographics, clinical aspects, laboratory/microbiology investigations, and patient clinical outcomes. Patients tested between October 2015 and January 2020 in King Abdulaziz Medical City, Riyadh, Saudi Arabia (KAMC-RD) were included. KAMC-RD is a tertiary care center with advance ICU services, and a large transplant and hematology center with a bed-capacity of more than 1500 beds. Community-acquired infections were defined as pneumonia occurring outside of the hospital settings. Hospital-acquired pneumonia was defined as pneumonia that occurs 48 hours or more after admission to the hospital and did not appear to be incubating at the time of admission, as per Infections Disease Society of America guidelines [28]. *Specimen sampling and testing:* Specimens were collected from the nasopharynx; they were tested using the BIOFIRE FILMARRAY Respiratory 2 plus using a realtime nested multiplexed polymerase chain reaction test designed to simultaneously identify nucleic acids for 18 viruses (including HKU1, OC34, NL63, and 229E) and 4 bacterial organisms that commonly affect the respiratory tract. It performs nested multiplex PCR in two stages; the first stage includes a single large volume multiplexed reaction. The second stage includes individual reactions to detect products from the first stage. *Statistical analysis:* We reported categorical data as frequencies and percentages; means and proportions of the study participants were calculated to characterize the study participants, overall and in groups. The primary outcome variable was HCoV infections. To signify the severity of clinical presentation, recognize the seasonal variations, and determine the overall outcome of HCoVs infections, the study participants were divided into four groups based on their HCoV infection type. The four groups (OC43, NL63, 229E, and HKU1) were compared using the Chi-square or Fisher’s exact test for categorical factors and the ANOVA test or Kruskal-Wallis Test for continuous variables as appropriate. Moreover, the study participants were divided into four groups based on their age at diagnosis. The four groups (pediatric (age ≤ 14), young adult (14 < age ≤ 24), and adult (24 < age)) were compared using Chi square with respect to participants’ comorbidities’ occurrence. The level of significance was declared at α = 0.05. Statistical analysis was conducted using SAS 9.4 (SAS Institute Inc., Cary, NC, USA).

## Figures and Tables

**Table 1:** Patient demographics.

	OC43 N = 570	NL63 N = 233	229E N = 125	HKU1 N = 134	Total N = 1062	*p*-Value
Age Mean ± SD	10.4 ± 23.12	12.1 ± 23.60	26.5 ± 31.93	17.8 ± 28.05	13.6 ± 25.57	<0.0001
Asthma	136 (23.9%)	38 (16.3%)	25 (20.0%)	22 (16.4%)	221 (20.8%)	0.0508
COPD	18 (3.2%)	7 (3.0%)	7 (5.6%)	6 (4.5%)	38 (3.6%)	0.4595
Congenital Heart Disease	127 (22.3%)	67 (28.8%)	22 (17.6%)	31 (23.1%)	247 (23.3%)	0.0899
Diabetes	45 (7.9%)	22 (9.4%)	36 (28.8%)	19 (14.2%)	122 (11.5%)	<0.0001
Heart Failure	51 (8.9%)	22 (9.4%)	27 (21.6%)	18 (13.4%)	118 (11.1%)	0.0004
Hypertension	51 (8.9%)	26 (11.2%)	35 (28.0%)	21 (15.7%)	133 (12.5%)	<0.0001
Immunosuppressed status *	65 (11.4%)	36 (15.5%)	26 (20.8%)	27 (20.1%)	154 (14.5%)	0.0073
Inhaled steroids use	110 (19.3%)	36 (15.5%)	22 (17.6%)	21 (15.7%)	189 (17.8%)	0.5375

* defined as patients with malignancies and/or taking immunosuppressive medications.

**Table 2:** Factors of transmission of HCoV.

	OC43	NL63	229E	HKU1	Total	*p*-Value
Contact with flu patients within 14 days	235 (41.2%)	87 (37.3%)	27 (21.6%)	33 (24.6%)	382 (36.0%)	<0.0001
Recent hospitalization within 30 days	158 (27.7%)	58 (24.9%)	52 (41.6%)	33 (24.6%)	301 (28.3%)	0.0042
Community acquired	537 (94.2%)	209 (89.7%)	108 (86.4%)	115 (85.8%)	969 (91.2%)	0.0014
Hospital acquired *	33 (5.8%)	24 (10.3%)	17 (13.6%)	19 (14.2%)	93 (8.8%)	0.0014
ICU acquired **	1 (3.0%)	1 (4.2%)	1 (5.9%)	00	3 (3.2%)	0.8840

* Infections occurring after 48 hours of admission to the hospital. ** Infections occurring after 48 hours of admission the ICU.

**Table 3:** Clinical symptoms.

	OC43	NL63	229E	HKU1	Total	*p*-Value
Fever	438 (76.8%)	172 (73.8%)	91 (72.8%)	97 (72.4%)	798 (75.1%)	0.5701
Productive cough	336 (58.9%)	121 (51.9%)	57 (45.6%)	65 (48.5%)	579 (54.5%)	0.0112
Shortness of breath	305 (53.5%)	112 (48.1%)	65 (52.0%)	69 (51.5%)	551 (51.9%)	0.5786
Upper respiratory symptoms	278 (48.8%)	103 (44.2%)	46 (36.8%)	55 (41.0%)	482 (45.4%)	0.0575

**Table 4:** Co-detection of other viruses with HCoV infections.

	OC43	NL63	229E	HKU1	Total	*p*-Value
Detection	282 (26.55)	122 (11.49)	74 (6.97)	74 (6.97)	535 (50.38)	0.0517
Adenovirus	89 (15.6%)	24 (10.3%)	14 (11.2%)	28 (20.9%)	155 (14.6%)	0.0250
Human Rhinovirus	126 (22.1%)	72 (30.9%)	30 (24.0%)	41 (30.6%)	269 (25.3%)	0.0288
Human metapneumo virus	25 (4.4%)	2 (0.9%)	1 (0.8%)	6 (4.5%)	34 (3.2%)	0.0123
Influenza A	18 (3.2%)	10 (4.3%)	5 (4.0%)	4 (3.0%)	37 (3.5%)	0.7933
Influenza B	9 (1.6%)	3 (1.3%)	1 (0.8%)	0	13 (1.2%)	0.6774
Parainfluenza virus 1	4 (0.7%)	5 (2.1%)	1 (0.8%)	0	10 (0.9%)	0.1783
Parainfluenza virus 2	0	1 (0.4%)	3 (2.4%)	2 (1.5%)	6 (0.6%)	0.0015
Parainfluenza virus 3	13 (2.3%)	4 (1.7%)	1 (0.8%)	5 (3.7%)	23 (2.2%)	0.4495
Parainfluenza virus 4	3 (0.5%)	2 (0.9%)	1 (0.8%)	3 (2.2%)	9 (0.8%)	0.2243
Respiratory syncytial virus	91 (16.0%)	27 (11.6%)	6 (4.8%)	22 (16.4%)	146 (13.7%)	0.0058

**Table 5:** Clinical outcomes of HCoV infections.

	OC43	NL63	229E	HKU1	Total	*p*-Value
Hospitalization. Mean Days (SD)	17.4 ± 92.59	10.7 ± 25.63	29.4 ± 83.33	29.2 ± 131.83	18.8 ± 88.27	<0.0001
ICU admission	61 (10.7%)	36 (15.5%)	33 (26.4%)	27 (20.1%)	157 (14.8%)	<0.0001
Required invasive ventilation	32 (56.1%)	24 (68.6%)	23 (67.6%)	12 (44.4%)	91 (59.5%)	0.3220
30-day mortality	7 (1.2%)	5 (2.1%)	8 (6.4%)	4 (3.0%)	24 (2.3%)	0.0081

## Data Availability

The datasets used and analyzed during this study are available from the corresponding author on reasonable request.
